# Exploring causal associations between the plasma proteome and bone mineral density: a Mendelian randomization study

**DOI:** 10.1530/EC-25-0180

**Published:** 2026-10-01

**Authors:** Kui Lv, Jialiu Fang, Shengyou Wang, Xing Xing, Rui Zhu

**Affiliations:** ^1^ Department of Emergency, Anhui No. 2 Provincial People’s Hospital, Hefei, Anhui, China; ^2^ Department of Orthopedics, The Second Affiliated Hospital of Anhui Medical University, Hefei, Anhui, China; ^3^ Department of Anesthesiology, Anhui Women and Children’s Medical Center/Hefei Maternal and Child Health Hospital, Hefei, Anhui, China; ^4^ Department of Biostatistics, Johns Hopkins Bloomberg School of Public Health, Baltimore, Maryland, USA

**Keywords:** circulating proteome, bone density, genetic, causal relationship, Mendelian randomization

## Abstract

**Introduction:**

Osteoporosis is characterized by reduced bone mineral density (BMD) and an increased risk of fractures, but the relationships between plasma proteins and site-specific BMD phenotypes remain unclear. We aimed to investigate the potential causal associations of plasma proteins with BMD using a Mendelian randomization (MR) analysis.

**Methods:**

We conducted a two-sample MR analysis as the primary analysis, using cis-protein quantitative trait locus (pQTL) instruments from nine proteomic genome-wide association studies and summary statistics for total-body, forearm, femoral-neck, and lumbar-spine BMD. Prioritized associations were evaluated using colocalization, Steiger directionality testing, protein-altering variant (PAV) annotation, cis-expression quantitative trait locus (cis-eQTL) overlap, and targeted replication. Protein–protein interaction (PPI), pathway enrichment, and druggability analyses were exploratory.

**Results:**

Twenty-three study-specific associations met the significance thresholds and involved 12 proteins: F2, TGFBI, PLEKHA1, HTRA1, PKDCC, CFHR4, LRP4, RSPO3, DKK1, TNFRSF11A, SPP1, and IBSP. Strong colocalization supported associations of F2 with total-body BMD, TGFBI with femoral-neck BMD, and PLEKHA1 with total-body and lumbar-spine BMD. Steiger testing supported directionality for 22 of 23 associations, except PKDCC–forearm BMD. Targeted replication provided no statistically significant, directionally concordant support. LRP4 relied on the only direct target-protein PAV, while eight cis-pQTLs for six proteins overlapped with cis-eQTLs. Exploratory analyses implicated skeletal and extracellular matrix processes and targets of prior pharmacological interest.

**Conclusion:**

Using cis-pQTL-based MR, we identified circulating proteins associated with BMD phenotypes. Strong colocalization was observed for F2, TGFBI, and PLEKHA1, whereas the remaining associations require additional validation. These findings provide genetic support for established bone-related pathways and highlight candidate proteins.

## Introduction

Osteoporosis is a prevalent skeletal disorder characterized by compromised bone strength and an increased susceptibility to fractures (1, 2). An estimated 18.5% of adults worldwide are affected by osteoporosis, with the disease disproportionately affecting older adults, particularly postmenopausal women, and imposing substantial health and economic burdens (3). Bone mineral density (BMD) is widely used to assess osteoporosis and serves as an important surrogate endpoint in therapeutic studies (4). Bone health and BMD are influenced by multiple factors, including aging, hormonal changes, diet, and physical activity (2, 5). Because reduced BMD may remain clinically asymptomatic until a fracture occurs, individuals without symptoms may not undergo imaging-based assessment. Moreover, differences between imaging scanners can introduce variability into longitudinal BMD measurements (4, 6). These factors may hinder the early identification of individuals with reduced BMD. Therefore, a better understanding of the factors associated with BMD may facilitate earlier risk identification and inform the prevention and management of osteoporosis.

Among the factors potentially associated with BMD, plasma proteins are of particular interest because they participate in a wide range of biological processes and may serve as biomarkers or therapeutic targets (7). Recent advances in large-scale proteomic and genome-wide association studies (GWASs) have enabled the identification of genetic variants associated with protein levels, referred to as protein quantitative trait loci (pQTLs) (8, 9). These variants can be used as instrumental variables (IVs) in Mendelian randomization (MR) analyses to investigate potential causal relationships between exposures and outcomes while reducing susceptibility to confounding and reverse causation (10).

Previous proteome-based genetic studies have investigated the relationships between circulating proteins and BMD. Han *et al.* evaluated 2,889 proteins in relation to estimated heel BMD and total-body BMD using two-sample MR, whereas Wang *et al.* used summary-data-based MR and colocalization analyses based on a single large pQTL resource and one BMD GWAS (11, 12). Building on these studies, we integrated genetic association data for circulating proteins from nine proteomic GWASs and outcome data from two large-scale BMD GWAS meta-analyses encompassing total-body, forearm, femoral-neck, and lumbar-spine BMD. We restricted the genetic instruments to cis-pQTLs and incorporated complementary analyses to assess cross-study consistency, causal directionality, colocalization, protein-altering variants (PAVs), and regulatory annotations. Therefore, this study aimed to evaluate genetically predicted associations between circulating proteins and BMD and to prioritize candidate proteins for further investigation in the context of osteoporosis.

## Methods

This study employed a two-sample MR framework to evaluate the associations between genetically predicted circulating protein levels and four DXA-derived BMD phenotypes measured at different skeletal sites (Fig. 1). Cis-acting pQTLs (cis-pQTLs), represented by single nucleotide polymorphisms (SNPs), were used as IVs. The primary cis-pQTL-based MR analysis was supplemented by colocalization analysis, Steiger directionality testing, PAV assessment, evaluation of overlap between pQTLs and expression quantitative trait loci (eQTLs), and targeted replication using the Sun *et al.* dataset. Protein–protein interaction (PPI), pathway enrichment, and druggability analyses were conducted as exploratory analyses to characterize the biological relevance and potential therapeutic implications of the prioritized proteins. Because the study used publicly available databases and previously published data, it did not involve direct interaction with human participants and was therefore exempt from ethics approval and informed consent requirements, in accordance with applicable institutional and regulatory policies.

**Figure 1:**
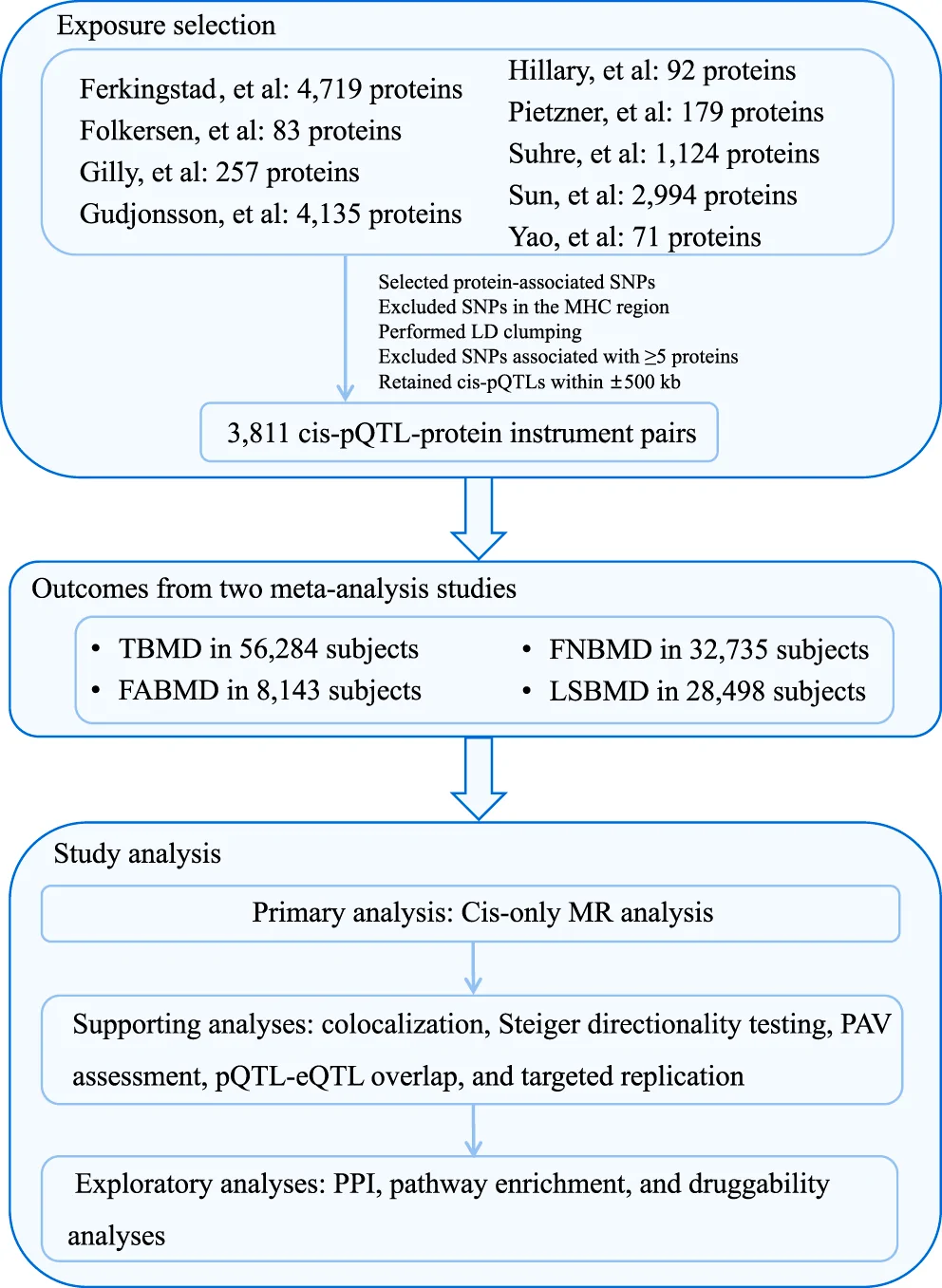
Flowchart of the Mendelian randomization analytical framework for circulating proteins and bone mineral density. TBMD, total-body bone mineral density; FABMD, forearm bone mineral density; FNBMD, femoral-neck bone mineral density; LSBMD, lumbar-spine bone mineral density; SNPs, single nucleotide polymorphisms; MHC, major histocompatibility complex; LD, linkage disequilibrium; pQTL, protein quantitative trait locus; eQTL, expression quantitative trait locus; PAV, protein-altering variant; PPI, protein–protein interaction.

### Study exposures

Human circulating proteins were treated as the exposure factors in this study. Summary statistics were obtained from nine GWASs, each of which included more than 500 samples and at least 50 quantified proteins (8, 13, 14, 15, 16, 17, 18, 19, 20). Across these nine studies, the combined reported sample size was 68,345, and the assay panels included 13,654 study-specific protein measurements. Instrument selection was performed in five sequential steps. First, eligible SNP–protein associations were defined according to the significance threshold specified in the original study (Supplementary Table 1 (see section on Supplementary materials given at the end of the article)), without applying an additional relaxed threshold (21). Second, variants located within the major histocompatibility complex (MHC) region on chromosome 6 (26–34 Mb) were excluded. Third, linkage disequilibrium (LD) clumping was performed separately for each protein using the Phase 3 European-ancestry reference panel from the 1,000 Genomes Project. Variants with an *r*^2^ ≥ 0.01 relative to the lead variant within a 500-kb window were removed. Fourth, variants associated with five or more proteins were excluded to reduce the potential influence of horizontal pleiotropy. Fifth, the remaining instruments were classified according to their genomic positions relative to the corresponding protein-encoding genes. The first four steps yielded 8,285 pQTL–protein instrument pairs, comprising 3,811 cis-pQTL pairs and 4,474 trans-pQTL pairs. Only cis-pQTL instruments were retained for the primary analysis. These were defined as variants located within ±500 kb of the corresponding protein-encoding gene. After harmonization according to UniProt accession numbers and restriction to proteins with at least one usable cis-pQTL, 1,558 unique proteins were included in the MR analysis. Among these proteins, 781 were represented in a single exposure study, whereas 777 were represented in two or more studies.

### Study outcomes

Summary statistics for total-body BMD (TBMD; *n* = 56,284) were obtained from the GWAS meta-analysis conducted by Medina-Gomez *et al.* (22). Summary statistics for forearm BMD (FABMD; *n* = 8,143), femoral-neck BMD (FNBMD; *n* = 32,735), and lumbar-spine BMD (LSBMD; *n* = 28,498) were obtained from the GWAS meta-analysis conducted by Zheng *et al.* (23). The four BMD phenotypes were analyzed separately and were not combined in a single meta-analysis.

### MR analysis

The primary analysis used cis-pQTLs as IVs for plasma proteins, with the four BMD phenotypes analyzed separately as outcomes. After matching the instruments to each outcome GWAS and harmonizing the effect alleles, the Wald ratio was used for protein–study–outcome combinations with a single available cis-pQTL. For combinations with two or more available instruments, a multiplicative random-effects inverse-variance weighted (IVW) analysis was performed using the mr_ivw function in the TwoSampleMR package. The choice of MR method was otherwise independent of the number of available instruments. Because the initial quality-control procedure could retain variants associated with two to four proteins, residual pleiotropy could not be completely excluded. Therefore, for protein–BMD associations that reached the multiple-testing-corrected significance threshold, we performed a more stringent pleiotropy sensitivity analysis. In this analysis, any remaining instrument associated with another measured protein was excluded, and the MR analysis was repeated using only the remaining protein-specific instruments. For protein–outcome comparisons involving at least three instruments and showing evidence of heterogeneity according to Cochran’s Q test, the weighted-median method was additionally applied as a sensitivity analysis. Potential residual locus-specific pleiotropy was further evaluated in subsequent analyses. Because separate MR estimates were generated when the same protein was measured in different exposure studies, the number of protein-study tests performed for each outcome was used to determine the Bonferroni correction. Accordingly, the significance threshold was calculated as 0.05 divided by the number of proteins tested (Supplementary Table 2). All analyses, including data harmonization, were performed using the TwoSampleMR R package (version 0.6.22; available at https://github.com/MRCIEU/TwoSampleMR). For proteins represented in more than one exposure study, study-specific MR estimates were additionally assessed for directional consistency. Concordant effect directions across exposure studies were interpreted as evidence of cross-study consistency rather than as formal independent replication.

### Colocalization analysis

Bayesian colocalization analysis was subsequently performed for protein–BMD associations that reached the multiple-testing-corrected significance threshold in the MR analyses. The purpose was to determine whether the protein and BMD associations were driven by a shared causal variant at a given genomic locus rather than by correlated signals arising from potentially complex LD (24). Colocalization analyses were conducted using the coloc R package (version 5.2.3; https://cran.r-project.org/web/packages/coloc). The analysis evaluated five hypotheses (H0–H4) and estimated the posterior probability for each hypothesis using the default prior probabilities (*P*_1_ = 1 × 10^−4^, *P*_2_ = 1 × 10^−4^, and *P*_12_ = 1 × 10^−5^). H4 represents the hypothesis that the same causal variant influences both the plasma protein and BMD. A posterior probability for H4 (PP.H4) > 0.80 was considered strong evidence of colocalization. To account for genomic regions containing multiple association signals, coloc.susie was additionally applied, with posterior probabilities reported for each exposure–outcome signal pair (25). For each tested cis-region, the number of SNPs available in both datasets after genome-build alignment, data harmonization, allele matching, and quality control was recorded as N_shared_SNPs. Where SuSiE identified no credible set for one or both traits, only the coloc.abf result is reported.

### Steiger filtering analysis

We performed the Steiger directionality test using the steiger_filtering function implemented in the TwoSampleMR R package to assess the inferred direction of association between the plasma proteins and BMD. A ‘true’ result was interpreted as supporting the hypothesized protein-to-BMD direction, whereas a ‘false’ result indicated that this direction was not supported. Importantly, a ‘false’ result was not interpreted as evidence of reverse causation. A nominal significance threshold of *P* < 0.05 was applied because the test was used solely to assess the directionality of the associations.

### PAV assessment

PAVs are genetic variants that alter the amino acid sequence of a protein and may consequently affect its structure, function, or expression. To assess potential interference from PAVs, we determined whether the cis-pQTLs used as IVs were themselves PAVs or were in LD with PAVs (*r*^2^ ≥ 0.8). The IVs were annotated using the Ensembl Variant Effect Predictor (VEP; release 115; https://asia.ensembl.org/Tools/VEP).

### Overlap of pQTLs and eQTLs

Overlap between pQTLs and eQTLs was assessed to determine whether the same genetic loci influence both protein abundance and mRNA expression, thereby improving the biological interpretability of the pQTLs used in the MR analysis. For each cis-pQTL supported by the MR analysis, the index variant and its proxies (*r*^2^ ≥ 0.8) were queried across all 49 tissues available in GTEx v8, regardless of their presumed relevance to bone metabolism or BMD. The analysis used precomputed significant cis-eQTL variant–gene pairs (signif_variant_gene_pairs; Version 8; https://www.gtexportal.org). These pairs were defined by GTEx using a 5% false discovery rate for eGenes and tissue- and gene-specific nominal *P*-value thresholds for variant–gene associations. No additional *P*-value threshold was imposed.

### Targeted replication analysis

To assess the robustness of the primary findings, targeted replication was performed for protein–BMD associations that reached statistical significance in the primary cis-MR analysis, using the 2023 UK Biobank plasma proteomics dataset reported by Sun *et al.* (7). The selected genetic instruments were harmonized with the four BMD GWAS datasets, and MR analyses were conducted using the Wald ratio for associations involving a single instrument and the IVW method for associations involving multiple instruments. After Bonferroni correction, statistical significance was defined as *P* < 0.005.

### PPI and gene enrichment analysis

To explore potential biological interactions among proteins identified across the four BMD outcomes, a PPI network was constructed using the STRING database (version 11.5; https://string-db.org/) with a minimum required interaction score of 0.4. No MR effect estimates or *P*-values were combined across outcomes. In addition, the functions of the corresponding protein-coding genes were characterized using Gene Ontology (GO) and Kyoto Encyclopedia of Genes and Genomes (KEGG) enrichment analyses. A *P*-value <0.05 was considered indicative of statistical significance. GO and KEGG enrichment analyses were performed using the ClusterProfiler R package (version 4.5; https://bioconductor.org/packages/release/bioc/html/clusterProfiler.html).

### Mapping of the MR-identified proteins with drug targets

To determine whether the MR-prioritized proteins overlapped with the druggable genome, we mapped them to the druggable gene set defined by Finan *et al.*, which comprises 4,479 genes classified into four tiers: Tier 1 (approved or clinical targets), Tier 2 (targets with bioactive ligands), Tier 3A (genetically supported or extracellular targets), and Tier 3B (other potentially druggable genes) (26). For each overlapping protein, we recorded its corresponding druggability tier and the available evidence for targeting or predicted targeting by small molecules or biotherapeutics. The MR-prioritized proteins were further annotated using the therapeutic target database (TTD; https://db.idrblab.net/ttd/), which contains 3,578 drug targets classified as successful, clinical, preclinical, or research targets, along with information on associated drugs and therapeutic indications.

## Results

### Determining the plasma proteins of genetic instruments

After quality-control filtering and restriction to cis-pQTLs, 3,811 cis-pQTL–protein instrument pairs were retained, corresponding to 2,958 exposure-study-specific protein measurements and 1,558 unique proteins. Following outcome-specific variant matching and allele harmonization, 3,650, 3,607, 3,633, and 3,634 instrument pairs were tested for TBMD, FABMD, FNBMD, and LSBMD, respectively. These analyses included 2,842, 2,819, 2,837, and 2,838 protein–study combinations, representing 1,534, 1,522, 1,530, and 1,530 unique proteins, respectively (Fig. 1; Supplementary Table 3).

### MR associations of plasma proteins with BMD

After outcome-specific Bonferroni correction, 23 significant exposure-study-specific cis-MR associations were prioritized. These associations represented 18 unique protein–BMD outcome pairs involving 12 unique proteins. Among the 23 significant associations, 18 were supported by a single cis-pQTL and 5 by two cis-pQTLs, yielding 28 instrument instances corresponding to 16 distinct cis-pQTLs (Fig. 2; Table 1; Supplementary Table 4). By outcome, TBMD was significantly associated with DKK1, TNFRSF11A, F2, LRP4, RSPO3, SPP1, IBSP, PLEKHA1, PKDCC, and HTRA1. FABMD was associated with PKDCC and CFHR4, whereas FNBMD was associated with TNFRSF11A, PKDCC, and TGFBI. LSBMD was associated with TNFRSF11A, SPP1, and PLEKHA1. Among the four BMD phenotypes, TBMD had the largest number of significant protein associations.

**Figure 2:**
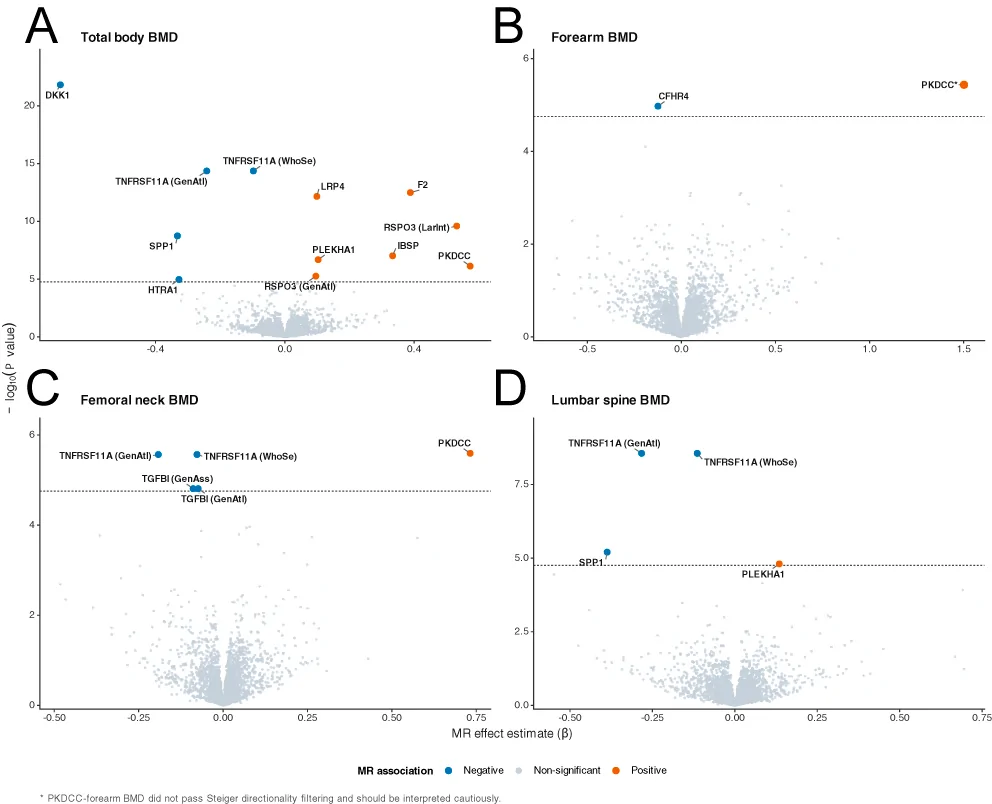
Cis-pQTL Mendelian randomization of circulating proteins and BMD: (A) total-body BMD, (B) forearm BMD, (C) femoral-neck BMD, and (D) lumbar-spine BMD. BMD, bone mineral density. One estimate per protein-study MR analysis; dashed lines show outcome-specific Bonferroni thresholds. PKDCC–forearm BMD did not pass Steiger directionality filtering and should be interpreted cautiously.

**Table 1:** Protein–phenotype associations identified by Mendelian randomization using cis-only pQTLs.

Uniport	Protein	Outcomes	Direction
O75096	LRP4 (14)	TBMD	Positive
O94907	DKK1 (14)	TBMD	Negative
P00734	Prothrombin (F2) (14)	TBMD	Positive
P10451	Osteopontin (SPP1) (14)	TBMD, LSBMD	Negative
P21815	IBSP (14)	TBMD	Positive
Q15582	TGFBI (7, 8)	FNBMD	Negative
Q504Y2	Extracellular tyrosine-protein kinase PKDCC (PKDCC) (14)	TBMD, FABMD, FNBMD	Positive
Q92496	CFHR4 (8)	FABMD	Negative
Q92743	Serine protease HTRA1 (HTRA1) (14)	TBMD	Negative
Q9BXY4	RSPO3 (7, 14)	TBMD	Positive
Q9HB21	PLEKHA1 (14)	TBMD, LSBMD	Positive
Q9Y6Q6	TNFRSF11A (7, 20)	TBMD, FNBMD, LSBMD	Negative

TBMD, total-body bone mineral density; FABMD, forearm bone mineral density; FNBMD, femoral-neck bone mineral density; LSBMD, lumbar-spine bone mineral density.

LRP4, low-density lipoprotein receptor-related protein 4; DKK1, Dickkopf-related protein 1; IBSP, integrin-binding sialoprotein; TGFBI, transforming growth factor-beta-induced protein ig-h3; CFHR4, complement factor H-related protein 4; RSPO3, R-spondin-3; PLEKHA1, pleckstrin homology domain-containing family A member 1; TNFRSF11A, tumor necrosis factor receptor superfamily member 11A.

Because all significant comparisons were supported by only one or two instruments, weighted-median analyses were not applicable to these associations. For nonsignificant comparisons, weighted-median analyses were additionally performed when at least three instruments were available and evidence of heterogeneity was detected. In total, 52 such comparisons were evaluated; however, none reached the corresponding outcome-specific Bonferroni-corrected significance threshold (Supplementary Table 5).

### Colocalization of pQTLs with BMD loci

To reduce potential bias arising from LD, Bayesian colocalization analysis was performed to determine whether protein and BMD associations were driven by a shared causal variant. Among the 28 cis-MR-prioritized associations identified using cis-pQTLs, seven showed strong evidence of colocalization (Supplementary Table 6). At the protein level, strong evidence supporting a shared causal variant was observed for F2, TGFBI, and PLEKHA1. The remaining prioritized proteins did not meet the predefined PP.H4 > 0.80 threshold and were therefore interpreted as putatively associated proteins or potential causal candidates rather than definitively causal proteins. Across the 23 tested protein–study–outcome regions, the number of shared variants ranged from 205 to 3,537 SNPs (median, 2,834). Region-specific values are provided separately from the number of independent signals identified by SuSiE in Supplementary Table 6.

### Assessment of directionality using Steiger filtering

Steiger filtering supported the inferred protein-to-BMD direction for 11 of the 12 prioritized proteins and for 22 of the 23 significant study-specific associations (Supplementary Table 7). The PKDCC–FABMD association, based on data from Ferkingstad *et al.*(14), did not pass the Steiger directionality test and was therefore classified as directionally uncertain. This finding may reflect reverse causation or differences in the proportion of variance explained by the instruments for the exposure and outcome, including potential measurement-related differences. In contrast, the associations of PKDCC with TBMD and FNBMD remained directionally supported.

### PAV investigation for the cis-pQTLs

Among the variants initially identified in the PAV assessment, rs2306029 was confirmed to be a missense variant in LRP4 and therefore represented a direct PAV of the measured target protein. In contrast, rs72959041 and rs7413610 were not direct PAVs of RSPO3 and CFHR4, respectively; instead, they were characterized as regulatory variants with supporting expression-related evidence (Supplementary Table 8). Because direct PAVs may influence measured protein levels through biological effects, assay-binding artifacts, or both, we assessed the robustness of the findings using independent non-PAV cis instruments where available. The LRP4–TBMD association could not be re-estimated after excluding rs2306029 because no alternative non-PAV cis instrument was available. For RSPO3, the independent non-PAV cis instruments produced effects in the same direction as the primary analysis. In contrast, the corresponding instruments for CFHR4 produced a nonsignificant effect in the opposite direction.

### Integrating the overlap of pQTLs and eQTLs

Using the precomputed significant cis-eQTL variant–gene pairs from GTEx v8 across all 49 tissues, eight cis-pQTLs corresponding to LRP4, SPP1, TGFBI, PKDCC, CFHR4, and RSPO3 overlapped with significant eQTL signals for the genes encoding the corresponding proteins (Supplementary Table 9). The tissues reported in Supplementary Table 9 were those in which matching significant eQTLs were identified and were not selected *a priori*. These overlaps were interpreted as evidence of potential transcription-mediated regulation rather than as evidence that any particular tissue is necessarily involved in the observed protein–BMD associations.

### Targeted replication analysis

Among the 12 significant proteins, seven (DKK1, TNFRSF11A, F2, RSPO3, SPP1, CFHR4, and TGFBI) had at least one usable Sun 2023 cis-pQTL, comprising 29 instruments. Five proteins (LRP4, IBSP, PLEKHA1, PKDCC, and HTRA1) could not be evaluated because no Sun 2023 cis instrument was available. Of the 18 originally significant protein–outcome pairs, 10 were therefore testable. None of the tested associations showed statistically significant replication with an effect direction concordant with the primary findings. TNFRSF11A–FNBMD (*β* = −0.111, SE = 0.176, *P* = 0.528) and SPP1–TBMD (*β* = −0.094, SE = 0.111, *P* = 0.394) showed directionally consistent but statistically nonsignificant effects. The remaining eight tested associations had effect estimates in the opposite direction from those observed in the primary analysis. Although F2–TBMD, RSPO3–TBMD, and TGFBI–FNBMD met the numerical replication *P*-value threshold, their effect directions were discordant with the primary findings; therefore, they were not considered successful replications. These replication results, together with the primary cis-MR findings and subsequent assessments of the 12 prioritized proteins, are summarized in Supplementary Table 13.

### Assessment of PPI and pathway enrichment analysis

An exploratory PPI analysis of the 12 proteins identified through the cis-pQTL MR analysis generated a network comprising 12 nodes and 9 edges. Given the small and biologically selected set of proteins, the observed interactions and enrichment of bone-related pathways should be interpreted as descriptive consistency with established bone biology rather than as evidence of newly identified biological pathways (Supplementary Fig. 1).

GO pathway enrichment analysis identified several pathways relevant to BMD, including ossification, bone resorption, integrin binding, and positive regulation of the noncanonical Wnt signaling pathway among the proteins prioritized by the cis-MR analysis (Supplementary Table 10; Supplementary Fig. 2). KEGG pathway analysis showed more limited enrichment among the cis-MR-prioritized proteins, with significant enrichment observed for the ECM-receptor interaction and complement and coagulation cascades pathways (Supplementary Table 11; Supplementary Fig. 3).

### Exploring drug targets for MR-selected proteins

Using the druggable gene database developed by Finan *et al.*, we assessed the MR-prioritized proteins for potential druggability. Of the 12 unique proteins identified in the MR analysis, 9 were included in the druggable gene set, comprising 3 Tier 1 targets, 5 Tier 3A targets, and 1 Tier 3B target (Table 2; Supplementary Table 12). Subsequently, analysis using the TTD identified six unique proteins as current or potential drug targets, including one successful target, two targets currently in clinical trials, and three targets supported by published literature. All six proteins also overlapped with the druggable targets identified in the Finan *et al.* dataset (Table 2; Supplementary Table 12).

**Table 2:** List of the MR-selected proteins that were drug targets or considered druggable.

Uniprot	Protein	Druggability tier	Target type
O94907	DKK1	Tier 1	Clinical trial target
P00734	Prothrombin (F2)	Tier 1	Successful target
P10451	Osteopontin (SPP1)	Tier 1	Literature-reported target
P21815	IBSP	Tier 3A	/
Q15582	TGFBI	Tier 3B	/
Q92496	CFHR4	Tier 3A	/
Q92743	Serine protease HTRA1 (HTRA1)	Tier 3A	Literature-reported target
Q9BXY4	RSPO3	Tier 3A	Clinical trial target
Q9Y6Q6	TNFRSF11A	Tier 3A	Literature-reported target

DKK1, Dickkopf-related protein 1; IBSP, integrin-binding sialoprotein; TGFBI, transforming growth factor-beta-induced protein ig-h3; CFHR4, complement factor H-related protein 4; RSPO3, R-spondin-3; TNFRSF11A, tumor necrosis factor receptor superfamily member 11A.

## Discussion

In this cis-pQTL-based two-sample MR analysis of four DXA-derived BMD phenotypes, 23 exposure-study-specific associations met the outcome-specific Bonferroni-corrected significance thresholds, involving 12 circulating proteins and 28 cis-pQTL instrument instances. The prioritized proteins included F2, TGFBI, and PLEKHA1, all of which were supported by strong colocalization evidence, as well as HTRA1. Other prioritized proteins included those that have been more extensively investigated in bone biology, such as DKK1, RSPO3, TNFRSF11A, SPP1, and IBSP, and proteins for which the available evidence was less consistent or more limited, including PKDCC, CFHR4, and LRP4. Overall, these findings genetically prioritize several circulating proteins for further investigation, while the complementary analyses provide additional context for interpreting the observed associations.

The distribution of prioritized associations varied across the four BMD phenotypes. TBMD accounted for the majority of prioritized associations, whereas FABMD, FNBMD, and LSBMD yielded fewer and partially overlapping associations. TBMD reflects bone density across the skeleton, whereas the site-specific phenotypes represent anatomically distinct skeletal regions. The one-third radius is predominantly cortical, the lumbar vertebral body is relatively rich in trabecular bone, and the proximal femur contains substantial contributions from both cortical and trabecular compartments (27). These anatomical differences may partly explain the observed patterns. However, differences in sample size, statistical power, and GWAS design limit the ability to draw definitive conclusions regarding site-specific effects.

Strong colocalization evidence further prioritized the associations of F2 with TBMD, TGFBI with FNBMD, and PLEKHA1 with TBMD and LSBMD. Among these proteins, F2 and PLEKHA1 were also identified by Han *et al.*, with directionally concordant associations across related BMD phenotypes (12). Higher genetically predicted circulating F2 levels were associated with greater TBMD. F2 encodes prothrombin, the precursor of thrombin, suggesting that coagulation-related signaling may contribute to skeletal remodeling. During fracture repair, thrombin activates the factor II thrombin receptor (F2R), also known as protease-activated receptor 1 (PAR1), in osteoblastic cells and regulates local inflammatory responses (28). Complementing these findings, Zhang *et al.* reported that F2R is highly expressed in osteoclasts and negatively regulates osteoclast formation and bone resorption (29). Consistent with these experimental observations, a clinical study of individuals with hemophilia found that higher thrombin generation was associated with higher BMD Z-scores (30). Collectively, these findings support the biological plausibility of the positive F2–TBMD association observed in our study. TGFBI, also known as BIGH3, was negatively associated with FNBMD. This inverse association is consistent with experimental studies of renal cell carcinoma bone metastasis, which showed that tumor-derived BIGH3 inhibits osteoblast differentiation and matrix mineralization while promoting osteocyte apoptosis and connexin-43-mediated gap-junction dysfunction (31, 32). These findings provide biological plausibility for the observed association, although the available experimental evidence was derived from tumor-associated osteolysis rather than BMD variation in the general population. PLEKHA1 was positively associated with TBMD and LSBMD, extending previous genetic evidence implicating the PLEKHA1 locus in FNBMD and type 2 diabetes and complementing bone-biopsy transcriptomic evidence linking PLEKHA1 to osteoporosis-associated gene networks (33). Together with the strong colocalization evidence observed for both associations, these findings further support the potential skeletal relevance of PLEKHA1. However, its direct functional role in bone biology remains unclear.

Another noteworthy finding was the inverse association between HTRA1 and TBMD, with higher genetically predicted circulating HTRA1 levels associated with lower TBMD. Osteoclast-derived HTRA1 degrades osteoprotegerin, thereby weakening its inhibitory effect on receptor activator of nuclear factor-κB ligand (RANKL)-dependent osteoclastogenesis (34). In a genetically modified mouse model, reduced HTRA1 expression was associated with enhanced osteoblastogenesis and attenuated age-related trabecular bone loss (35). These experimental findings are consistent with the inverse association observed in our study. However, HTRA1 may exert context-dependent effects on bone remodeling. HTRA1-mediated cleavage of thrombospondin-1 can release a fragment that inhibits osteoclast activity, whereas reduced HTRA1 expression has also been observed in human osteoporotic bone (36, 37). Taken together, these findings suggest that the skeletal effects of HTRA1 may depend on its specific substrates and local tissue environment.

The findings for PKDCC, CFHR4, and LRP4 warrant cautious interpretation because of uncertainties regarding causal directionality and potential assay-related effects. PKDCC was positively associated with FABMD, FNBMD, and TBMD in our study. Maridas *et al.* showed that conditional deletion of PKDCC in mouse skeletal progenitors reduced bone volume and delayed fracture repair (38). Complementary genetic and functional evidence has linked the regulation of PKDCC expression in chondrocytes to craniofacial skeletal development (39). These findings provide biological support for a potential role of PKDCC in skeletal biology and are broadly compatible with our observed BMD associations. However, the PKDCC–FABMD association did not pass Steiger filtering, leaving the directionality of this association uncertain. CFHR4 is a regulator of the alternative complement pathway. Although its effects on BMD have not been experimentally established, complement factor H deficiency has been associated with impaired osteoblast differentiation and reduced bone mass, providing indirect evidence that complement regulation may influence bone biology (40). Wang *et al.* reported an association between genetically predicted CFHR4 levels and lower osteoporotic fracture risk; however, fracture risk and FABMD represent distinct skeletal outcomes (41). Moreover, the lack of support from alternative non-PAV instruments further limits the robustness of the CFHR4–FABMD association.

DKK1, RSPO3, and LRP4 are involved in Wnt/β-catenin signaling, a key pathway regulating osteoblast differentiation and bone formation. DKK1 was negatively associated with TBMD, whereas RSPO3 showed a positive association, broadly consistent with their respective inhibitory and potentiating effects on Wnt signaling (42, 43). The positive association between RSPO3 and BMD is also consistent with previous genetic evidence linking higher RSPO3 levels to greater BMD and lower fracture risk (12). In contrast, the positive LRP4-TBMD association observed in our study differs from experimental evidence indicating that LRP4 facilitates sclerostin-mediated inhibition of bone formation (44). Previous MR evidence for LRP4 is mixed, with Han *et al.* reporting an inverse association with BMD, whereas Zhang *et al.* prioritized LRP4 for osteoporosis (12, 45). The latter supports the skeletal relevance of LRP4 but does not directly confirm our finding because the outcomes differed. Moreover, the LRP4–TBMD association was supported by a direct PAV, which may introduce potential assay-related effects. Therefore, this association should be interpreted cautiously.

TNFRSF11A was inversely associated with FNBMD, LSBMD, and TBMD. TNFRSF11A encodes receptor activator of nuclear factor-κB (RANK), whose activation by RANKL promotes osteoclast differentiation and bone resorption (46). Recent evidence showing that endothelial RANK deletion protects mice against ovariectomy-induced bone loss further suggests that RANK signaling may influence skeletal homeostasis through the bone marrow vascular microenvironment (47). SPP1 and IBSP are well-characterized bone matrix proteins that contribute to bone mineralization and remodeling (48).

The follow-up analyses provided additional context for interpreting the primary MR findings. Colocalization strengthened the evidence for associations involving F2, TGFBI, and PLEKHA1 by supporting shared genetic signals. However, the absence of strong colocalization for the remaining associations may reflect limited statistical power or complex LD rather than necessarily excluding an underlying biological relationship (25). Steiger testing was generally consistent with the hypothesized protein-to-BMD direction, whereas the PKDCC–FABMD association remained directionally uncertain and did not provide evidence of reverse causation. Overlap with cis-eQTLs further supported a potential regulatory basis for several instruments, although such overlap does not establish transcriptional mediation of the corresponding BMD associations.

Instrument-level evaluation revealed additional differences in the robustness of the prioritized associations. The LRP4–TBMD association depended on a direct PAV, which may affect protein function, assay binding, or both. Because no alternative non-PAV cis instrument was available, these possibilities could not be distinguished. Similarly, alternative non-PAV instruments did not support the CFHR4 association, further weakening the robustness of this finding. Targeted replication using the Sun *et al.* (7) dataset also yielded no statistically significant replication with an effect direction concordant with the primary analysis. This lack of replication highlights the need for further validation across independent proteomic resources, particularly given potential differences in assay platforms, protein measurement characteristics, and the genetic instruments available across datasets.

Finally, exploratory interaction, pathway, and druggability analyses placed the prioritized proteins within skeletal and extracellular matrix-related processes and identified several proteins with prior pharmacological relevance. Given the small and selectively identified protein set, however, these findings should be interpreted as providing biological context rather than as evidence of shared causal mechanisms or therapeutic efficacy.

Several limitations of this study should be acknowledged. First, all prioritized protein–BMD associations were supported by only one or two cis-pQTLs, precluding conventional multi-instrument sensitivity analyses, such as the weighted-median and MR-Egger methods. Second, estimates for proteins measured across multiple exposure studies were not meta-analyzed, and pooled effect estimates were therefore not obtained. Third, although UK Biobank Pharma Proteomics Project (UKB-PPP) data were included in the targeted replication analysis, potential sample overlap with the BMD GWAS datasets remained a possible source of bias. Fourth, the cis-only design did not evaluate associations instrumented by trans-pQTLs. Fifth, the GTEx-based eQTL annotations provided regulatory context but could not establish which tissues or bone-cell populations contributed to the observed associations. Finally, the contributing populations were predominantly of European ancestry, which may limit the generalizability of the findings to other populations.

In conclusion, this cis-pQTL-based MR study prioritized 12 circulating proteins associated with four BMD phenotypes. Among these, the associations involving F2, TGFBI, and PLEKHA1 received additional support from colocalization analysis. Overall, the findings provide a focused set of candidate proteins for independent replication and further tissue-specific and functional investigation.

## Supplementary materials





## Declaration of interest

The authors declare that they have no conflicts of interest that could be perceived as prejudicing the impartiality of this research.

## Funding

This work was supported by the Natural Science Foundation of Anhui Medical University (No. 2023xkj105).

## Author contribution statement

KL contributed to the formal analysis, writing of the original draft, and review and editing of the manuscript. JF contributed to writing, review, and editing of the manuscript and project administration. SW performed data curation and validation. XX contributed to formal analysis, methodology, software, and writing, review, and editing of the manuscript. RZ contributed to conceptualization, supervision, validation, and writing, review, and editing of the manuscript. All authors reviewed and revised the draft, approved the final version, and accept accountability for its accuracy and integrity.

## Data availability

Data were obtained from publicly available open-access repositories, with plasma proteins derived from nine proteomic GWASs and BMD from two meta-analysis studies, as listed in Refs. 8, 13, 14, 15, 16, 17, 18, 19, 20, 22, 23.

## Ethics approval and consent to participate

This study utilized publicly available, ethically approved data with informed consent from subjects, eliminating the need for additional ethical approval or consent forms.

## References

[1] Foessl I, Dimai HP & Obermayer-Pietsch B. Long-term and sequential treatment for osteoporosis. Nat Rev Endocrinol 2023 19 520–533. (10.1038/s41574-023-00866-9) 37464088

[2] Jones BC, Lee H, Cheng CC, et al. MRI quantification of cortical bone porosity, mineralization, and morphologic structure in postmenopausal osteoporosis. Radiology 2023 307 e221810. (10.1148/radiol.221810) PMC10102628 36692396

[3] Chen S, Xu X, Gong H, et al. Global epidemiological features and impact of osteosarcopenia: a comprehensive meta-analysis and systematic review. J Cachexia Sarcopenia Muscle 2024 15 8–20. (10.1002/jcsm.13392) PMC10834350 38086772

[4] Black DM, Bauer DC, Vittinghoff E, et al. Treatment-related changes in bone mineral density as a surrogate biomarker for fracture risk reduction: meta-regression analyses of individual patient data from multiple randomised controlled trials. Lancet Diabetes Endocrinol 2020 8 672–682. (10.1016/s2213-8587(20)30159-5) 32707115

[5] Xu J, Cai X, Miao Z, et al. Proteome-wide profiling reveals dysregulated molecular features and accelerated aging in osteoporosis: a 9.8-year prospective study. Aging Cell 2024 23 e14035. (10.1111/acel.14035) PMC10861190 37970652

[6] Lee K, Al Jumaily K, Lin M, et al. Dual-energy x-ray absorptiometry scanner mismatch in follow-up bone mineral density testing. Osteoporos Int 2022 33 1981–1988. (10.1007/s00198-022-06438-3) 35614236

[7] Sun BB, Chiou J, Traylor M, et al. Plasma proteomic associations with genetics and health in the UK Biobank. Nature 2023 622 329–338. (10.1038/s41586-023-06592-6) PMC10567551 37794186

[8] Gudjonsson A, Gudmundsdottir V, Axelsson GT, et al. A genome-wide association study of serum proteins reveals shared loci with common diseases. Nat Commun 2022 13 480. (10.1038/s41467-021-27850-z) PMC8789779 35078996

[9] Boehm FJ & Zhou X. Statistical methods for Mendelian randomization in genome-wide association studies: a review. Comput Struct Biotechnol J 2022 20 2338–2351. (10.1016/j.csbj.2022.05.015) PMC9123217 35615025

[10] Skrivankova VW, Richmond RC, Woolf BAR, et al. Strengthening the reporting of observational studies in epidemiology using Mendelian randomization: the STROBE-MR statement. JAMA 2021 326 1614–1621. (10.1001/jama.2021.18236) 34698778

[11] Wang L, Guo X, Qin J, et al. Assessing the causal relationship between plasma proteins and osteoporosis: novel insights into pathological mechanisms and therapeutic implications. Osteoporos Int 2024 35 1973–1987. (10.1007/s00198-024-07225-y) 39120624

[12] Han B-X, Yan S-S, Yu H, et al. Causal effects of plasma proteome on osteoporosis and osteoarthritis. Calcif Tissue Int 2023 112 350–358. (10.1007/s00223-022-01049-w) 36576504

[13] Sun BB, Maranville JC, Peters JE, et al. Genomic atlas of the human plasma proteome. Nature 2018 558 73–79. (10.1038/s41586-018-0175-2) PMC6697541 29875488

[14] Ferkingstad E, Sulem P, Atlason BA, et al. Large-scale integration of the plasma proteome with genetics and disease. Nat Genet 2021 53 1712–1721. (10.1038/s41588-021-00978-w) 34857953

[15] Suhre K, Arnold M, Bhagwat AM, et al. Connecting genetic risk to disease end points through the human blood plasma proteome. Nat Commun 2017 8 14357. (10.1038/ncomms14357) PMC5333359 28240269

[16] Hillary RF, McCartney DL, Harris SE, et al. Genome and epigenome wide studies of neurological protein biomarkers in the Lothian Birth Cohort 1936. Nat Commun 2019 10 3160. (10.1038/s41467-019-11177-x) PMC6639385 31320639

[17] Pietzner M, Wheeler E, Carrasco-Zanini J, et al. Genetic architecture of host proteins involved in SARS-CoV-2 infection. Nat Commun 2020 11 6397. (10.1038/s41467-020-19996-z) PMC7744536 33328453

[18] Yao C, Chen G, Song C, et al. Genome-wide mapping of plasma protein QTLs identifies putatively causal genes and pathways for cardiovascular disease. Nat Commun 2018 9 3268. (10.1038/s41467-018-05512-x) PMC6093935 30111768

[19] Folkersen L, Fauman E, Sabater-Lleal M, et al. Mapping of 79 loci for 83 plasma protein biomarkers in cardiovascular disease. PLoS Genet 2017 13 e1006706. (10.1371/journal.pgen.1006706) PMC5393901 28369058

[20] Gilly A, Park YC, Png G, et al. Whole-genome sequencing analysis of the cardiometabolic proteome. Nat Commun 2020 11 6336. (10.1038/s41467-020-20079-2) PMC7729872 33303764

[21] Zhang Y, Xie J, Wen S, et al. Evaluating the causal effect of circulating proteome on the risk of osteoarthritis-related traits. Ann Rheum Dis 2023 82 1606–1617. (10.1136/ard-2023-224459) 37595989

[22] Medina-Gomez C, Kemp JP, Trajanoska K, et al. Life-course genome-wide association study meta-analysis of total body BMD and assessment of age-specific effects. Am J Hum Genet 2018 102 88–102. (10.1016/j.ajhg.2017.12.005) PMC5777980 29304378

[23] Zheng HF, Forgetta V, Hsu YH, et al. Whole‐genome sequencing identifies EN1 as a determinant of bone density and fracture. Nature 2015 526 112–117. (10.1038/nature14878) PMC4755714 26367794

[24] Zheng J, Haberland V, Baird D, et al. Phenome-wide Mendelian randomization mapping the influence of the plasma proteome on complex diseases. Nat Genet 2020 52 1122–1131. (10.1038/s41588-020-0682-6) PMC7610464 32895551

[25] Wallace C. A more accurate method for colocalisation analysis allowing for multiple causal variants. PLoS Genet 2021 17 e1009440. (10.1371/journal.pgen.1009440) PMC8504726 34587156

[26] Finan C, Gaulton A, Kruger FA, et al. The druggable genome and support for target identification and validation in drug development. Sci Transl Med 2017 9 eaag1166. (10.1126/scitranslmed.aag1166) PMC6321762 28356508

[27] Liu XS, Cohen A, Shane E, et al. Bone density, geometry, microstructure, and stiffness: relationships between peripheral and central skeletal sites assessed by DXA, HR-pQCT, and cQCT in premenopausal women. J Bone Miner Res 2010 25 2229–2238. (10.1002/jbmr.111) PMC3128822 20499344

[28] Sato N, Ichikawa J, Wako M, et al. Thrombin induced by the extrinsic pathway and PAR-1 regulated inflammation at the site of fracture repair. Bone 2016 83 23–34. (10.1016/j.bone.2015.10.005) 26475502

[29] Zhang Y, Wang H, Zhu G, et al. F2r negatively regulates osteoclastogenesis through inhibiting the Akt and NFκB signaling pathways. Int J Biol Sci 2020 16 1629–1639. (10.7150/ijbs.41867) PMC7097923 32226307

[30] Citla-Sridhar D, Chung S, Brown AW, et al. Bone mineral density, bone remodeling biomarkers, and hemostatic correlates in hemophilia and von Willebrand disease. Blood Adv 2026 10 5576–5586. (10.1182/bloodadvances.2026019657) PMC13487677 42166691

[31] Pan T, Lin SC, Yu KJ, et al. BIGH3 promotes osteolytic lesions in renal cell carcinoma bone metastasis by inhibiting osteoblast differentiation. Neoplasia 2018 20 32–43. (10.1016/j.neo.2017.11.002) PMC5711998 29190493

[32] Pan T, Liu F, Hao X, et al. BIGH3 mediates apoptosis and gap junction failure in osteocytes during renal cell carcinoma bone metastasis progression. Cancer Lett 2024 596 217009. (10.1016/j.canlet.2024.217009) PMC11964150 38849015

[33] Hu Y, Tan LJ, Chen XD, et al. Identification of novel variants associated with osteoporosis, type 2 diabetes and potentially pleiotropic loci using pleiotropic cFDR method. Bone 2018 117 6–14. (10.1016/j.bone.2018.08.020) PMC6364698 30172742

[34] Ochiai N, Nakachi Y, Yokoo T, et al. Murine osteoclasts secrete serine protease HtrA1 capable of degrading osteoprotegerin in the bone microenvironment. Commun Biol 2019 2 86. (10.1038/s42003-019-0334-5) PMC6397181 30854478

[35] Murayama M, Hirata H, Shiraki M, et al. Nupr1 deficiency downregulates HtrA1, enhances SMAD1 signaling, and suppresses age-related bone loss in male mice. J Cell Physiol 2023 238 566–581. (10.1002/jcp.30949) 36715607

[36] Carminati L, Sangalli F, Urbinati C, et al. TSP-1 interaction with RANK and OPG: implications for bone remodeling and osteolytic bone metastasis. Cell Death Dis 2026 17 332. (10.1038/s41419-026-08600-9) PMC13039915 41862435

[37] Licini C, Fantone S, Lamanna D, et al. Possible involvement of HtrA1 serine protease in the onset of osteoporotic bone extracellular matrix changes. Tissue Cell 2024 87 102329. (10.1016/j.tice.2024.102329) 38367326

[38] Maridas DE, Gamer L, Moore ER, et al. Loss of vlk in Prx1+ cells delays the initial steps of endochondral bone formation and fracture repair in the limb. J Bone Miner Res 2022 37 764–775. (10.1002/jbmr.4514) 35080046

[39] Mohammed J, Arora N, Matthews HS, et al. A common cis-regulatory variant impacts normal-range and disease-associated human facial shape through regulation of PKDCC during chondrogenesis. Elife 2024 13 e82564. (10.7554/elife.82564) PMC10939500 38483448

[40] Chen L, Hu H, Qiu W, et al. Complement factor H (CFH), an immune factor enhances osteoblast differentiation and correlates with bone density. J Bone Miner Res 2026 In press zjag095. (10.1093/jbmr/zjag095) 42301900

[41] Wang Q, Kuang Q, Chen C, et al. Genetically predicted KIR2DS4 mediate the association between gut microbe K10 and osteoporosis fractures: a mediation Mendelian randomization study. PM R 2026 18 426–442. (10.1002/pmrj.70029) PMC13081771 41230735

[42] Ko JY, Wang FS, Lian WS, et al. Dickkopf-1 (DKK1) blockade mitigates osteogenesis imperfecta (OI) related bone disease. Mol Med 2024 30 66. (10.1186/s10020-024-00838-3) PMC11106911 38773377

[43] Nilsson KH, Henning P, El Shahawy M, et al. RSPO3 is important for trabecular bone and fracture risk in mice and humans. Nat Commun 2021 12 4923. (10.1038/s41467-021-25124-2) PMC8363747 34389713

[44] Wang L, Tao X, Jiang H, et al. Osteoblastic sclerostin loop3-LRP4 interaction required by sclerostin to inhibit bone formation. Bone Res 2026 14 45. (10.1038/s41413-026-00511-x) PMC13109412 42031715

[45] Zhang Z, He J, Lin X, et al. Roles of circulating proteins in osteoporosis pathogenesis: insights from Mendelian randomization. Bone 2026 212 118005. (10.1016/j.bone.2026.118005) 42419582

[46] Li J, Sarosi I, Yan X-Q, et al. RANK is the intrinsic hematopoietic cell surface receptor that controls osteoclastogenesis and regulation of bone mass and calcium metabolism. Proc Natl Acad Sci U S A 2000 97 1566–1571. (10.1073/pnas.97.4.1566) PMC26475 10677500

[47] Kaneko T, Yari S, Kikuta J, et al. The RANK/RANKL axis controls vascular dynamics in the bone marrow. Proc Natl Acad Sci U S A 2025 122 e2425366122. (10.1073/pnas.2425366122) PMC12625855 41183210

[48] Malaval L, Follet H, Farlay D, et al. OPN, BSP, and bone quality – structural, biochemical, and biomechanical assessment in OPN−/−, BSP−/−, and DKO mice. Calcif Tissue Int 2024 115 63–77. (10.1007/s00223-024-01217-0) 38733411

