# Supplementary material for: Exploring causal associations between the plasma proteome and bone mineral density: a Mendelian randomization study
Source: Endocr Connect. 2026 Oct 1;15(10):e250180. doi: 10.1530/EC-25-0180 (PMC13663631; doi:10.1530/EC-25-0180)
Supplement: Supplementary file 1 [file EC-25-0180_supplementary_figures.pdf]

Supplementary figure legends

Supplementary Figure 1. Protein-protein interaction networks of the proteins identified by cis Mendelian randomization.

Supplementary Figure 2. Functional Gene Ontology (GO) pathway enrichment analysis of proteins identified in cis-Mendelian randomization. Bar plot (A) and bubble plot (B) show enriched biological pathways.

Supplementary Figure 3. Functional Kyoto Encyclopedia of Genes and Genomes (KEGG) pathway enrichment analysis of proteins identified in cis-Mendelian randomization. Bar plot (A) and bubble plot (B) show enriched biological pathways.

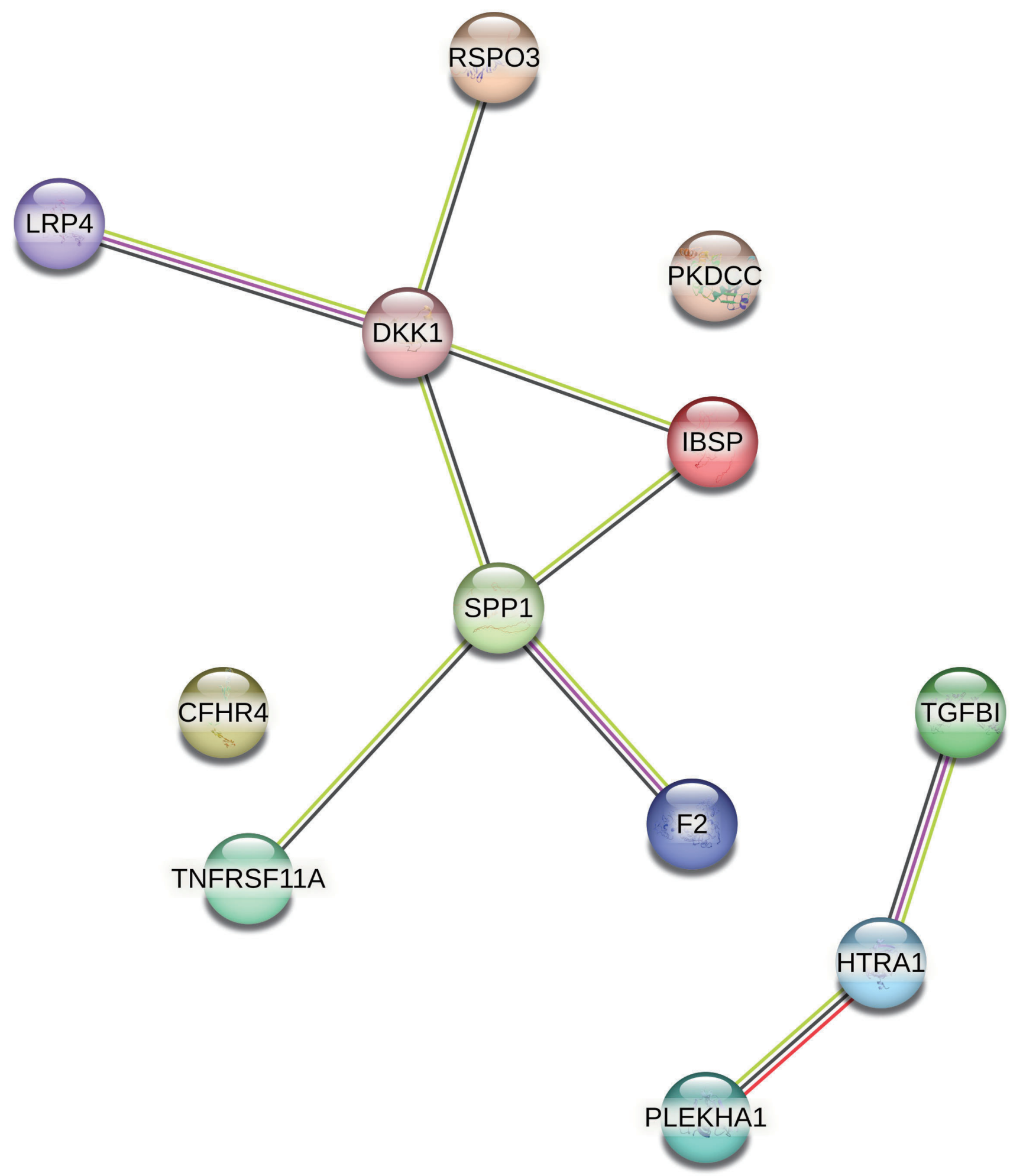

Supplementary Figure 1. Protein-protein interaction networks of the proteins identified by cis Mendelian randomization.

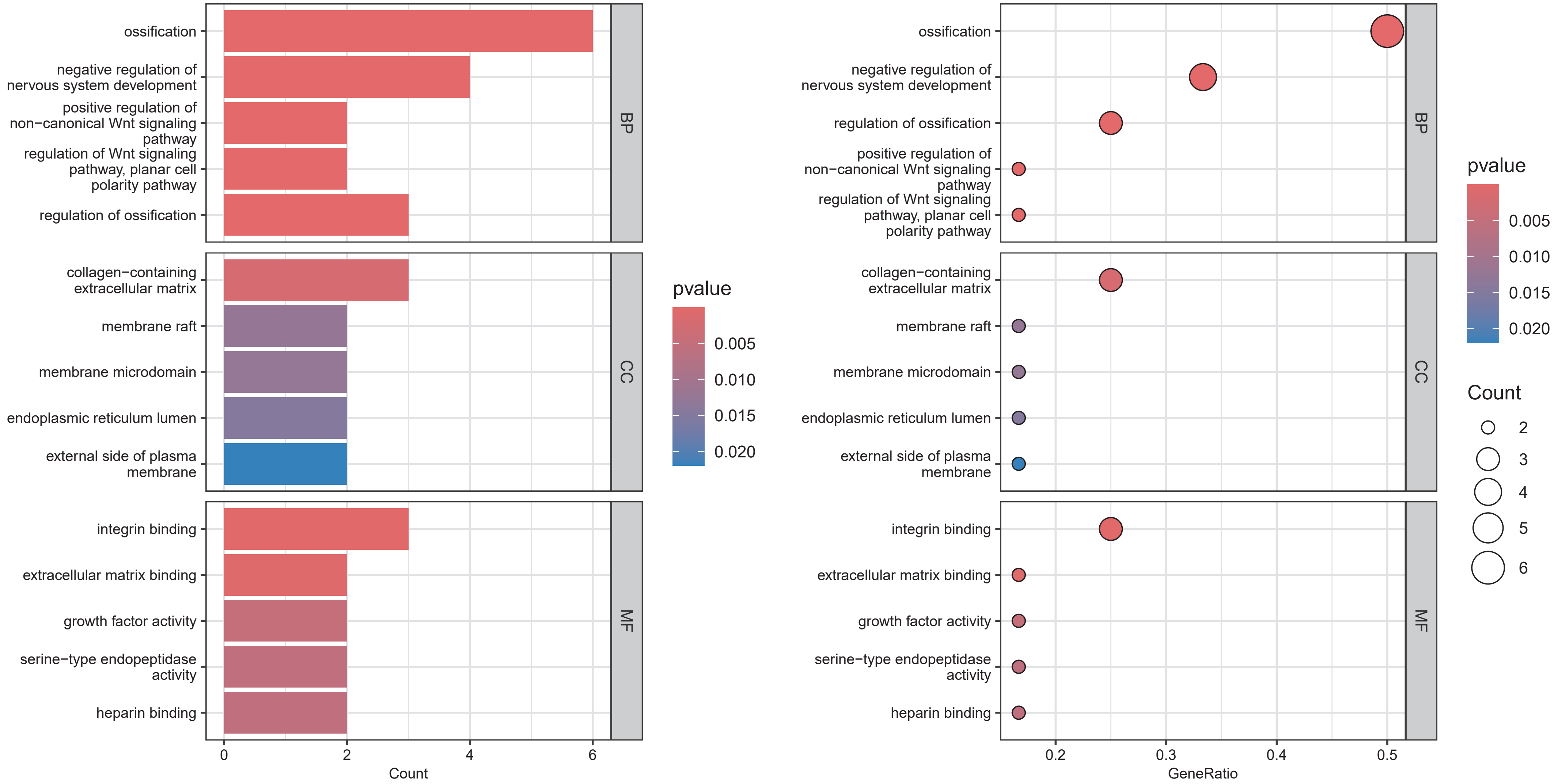

Supplementary Figure 2. Functional Gene Ontology (GO) pathway enrichment analysis of proteins identified in cis-Mendelian randomization. Bar plot (A) and bubble plot (B) show enriched biological pathways

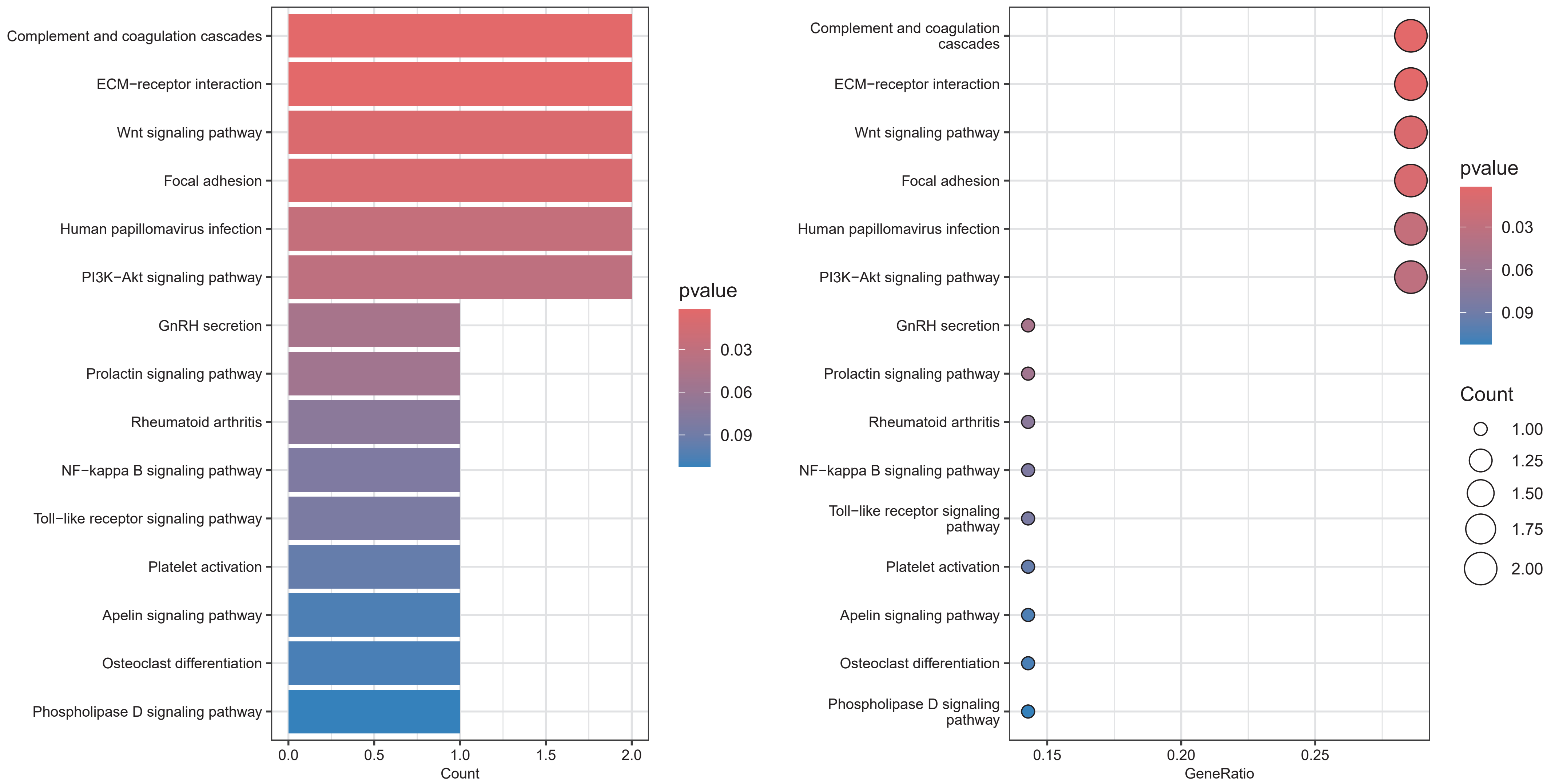

Supplementary Figure 3. Functional Kyoto Encyclopedia of Genes and Genomes (KEGG) pathway enrichment analysis of proteins identified in cis-Mendelian randomization. Bar plot (A) and bubble plot (B) show enriched biological pathways.
